# Heidelberger Interprofessionelle Ausbildungsstation (HIPSTA): a practice- and theory-guided approach to development and implementation of Germany’s first interprofessional training ward

**DOI:** 10.3205/zma001179

**Published:** 2018-08-15

**Authors:** André L. Mihaljevic, Jochen Schmidt, Anika Mitzkat, Pascal Probst, Theresa Kenngott, Johanna Mink, Christoph A. Fink, Alexej Ballhausen, Jessy Chen, Aylin Cetin, Lisa Murrmann, Gisela Müller, Cornelia Mahler, Burkhard Götsch, Birgit Trierweiler-Hauke

**Affiliations:** 1 Universitätsklinik Heidelberg, Klinik für Allgemein-, Viszeral und Transplantationschirurgie, Heidelberg, Germany; 2 Universitätsklinik Heidelberg, Pflegedienst Chirurgische Klinik und Klinik für Anästhesiologie, Heidelberg, Germany; 3 Universitätsklinik Heidelberg, Abteilung Allgemeinmedizin und Versorgungsforschung, Heidelberg, Germany; 4 Fachschaft Medizin Heidelberg, Heidelberg, Germany; 5 Akademie für Gesundheitsberufe Heidelberg, Gesundheits- und Krankenpflegeschule, Heidelberg, Germany; 6 Akademie für Gesundheitsberufe Heidelberg gGmbH, Gesundheits- und Krankenpflegeschule, Heidelberg, Germany

**Keywords:** Interprofessional relations, interprofessional education, interprofessional learning, interdisciplinary communication, interprofessional collaborative practice, interprofessional training ward, general surgery

## Abstract

**Background: **Deficits in care and impaired patient-safety have been linked to inefficient interprofessional collaborative practice. Interprofessional training wards (IPTW) are an interprofessional educational intervention which aim to enable students and trainees from different health professions to work self-responsibly in order to manage the medical treatment and rehabilitation of real-life patients together as an interprofessional team. We aimed to develop and implement Germany´s first IPTW at the department of Surgery at Heidelberg University Hospital.

**Methods: **The Kern cycle was used to develop an ITPW curriculum. Practical as well as theoretical considerations guided the design of the IPTW. Common project management tools including blueprinting and RASCI (Responsibility, Approval, Support, Consultation, Information) matrix were applied.

**Results: **Since April 2017, 7 cohorts of students and trainees have had four-week long placements on HIPSTA. They run the IPTW in early and late shifts. Nursing and medical facilitators are supporting the IP team as needed. Learning objectives are operationalized as EPAs (entrustable professional activities) and interprofessional learning goals. Since initiation only minor modifications to the curriculum have been necessary and satisfaction of students/trainees, facilitators and patients is high.

**Conclusion:** IPTWs can be established and run in the German health care system even in a complex clinical setting. The early involvement of all professions in a steering group seems to be key to success. Nursing and medical facilitators are of utmost importance for daily routine. The experiences outlined here could help others aiming to implement IPTWs at their sites. IPTWs might address a number of hitherto unaddressed educational needs.

**Trial registration: **Not applicable

## Zusammenfassung

**Hintergrund: **Versorgungsdefizite und eine Gefährdung der Patientensicherheit sind wiederholt mit Mängeln in der interprofessionellen kollaborativen Zusammenarbeit in Verbindung gebracht worden. Interprofessionelle Ausbildungsstationen (IPSTA) sind eine interprofessionelle Ausbildungsintervention, die Studierende und Auszubildende verschiedener Gesundheitsberufe in die Lage versetzen sollen, gemeinsam als Team eigenverantwortlich die Versorgung von Patienten im realen Stationsalltag zu leisten. Ziel unseres Projekts war die Entwicklung und Implementierung von Deutschlands erster IPSTA in der Abteilung für Allgemein-, Viszeral- und Transplantationschirurgie an der Universitätsklinik Heidelberg.

**Methoden: **Der Kern-Zyklus wurde benutzt, um eine IPSTA Curriculum zu entwickeln. Praktische wie auch theoretische Überlegungen wurden dabei berücksichtigt. Dabei wurden übliche Management-Werkzeuge wie das Blueprinting und die RASCI-Matrix (Responsibility, Approval, Support, Consultation, Information) genutzt.

**Ergebnisse: **Seit April 2017 hatten 7 Kohorten aus Medizinstudierenden und Auszubildenden der Gesundheits- und Krankenpflege 4-wöchige Einsätze auf der Heidelberger Interprofessionellen Ausbildungsstation (HIPSTA). Die interprofessionellen Teams betreuen die Patienten in zwei Schichten (Früh- und Spätschicht) unter Aufsicht von ärztlichen und pflegerischen Lernbegleitern. Ärztliche und pflegerische Lernbegleiter. Lernziele wurden durch interprofessionelle anvertraubare professionelle Tätigkeiten (entrusted professional activities) und interprofessionelle Lernziele operationalisiert. Seit Beginn mussten nur kleinere Veränderungen am HIPSTA Curriculum vorgenommen werden. Die Zufriedenheit von Patienten, der Studierenden und Auszubildenden und der Lernbegleiter ist hoch.

**Schlussfolgerung: **Interprofessionelle Ausbildungsstationen können im deutschen Gesundheitssystem auch in komplexen klinischen Umgebungen erfolgreich etabliert werden. Die frühe Einbindung aller beteiligter Professionen bereits in der Planungsphase scheint für den dauerhaften Erfolg essentiell zu sein. Ärztliche und pflegerische Lernbegleiter sind für den täglichen Ablauf unabdingbar. IPSTAs adressieren einen bisher nicht gedeckten Lernbedarf.

**Studienanmeldung:** Nicht zutreffend

## Introduction

Failure to deliver efficient interprofessional collaborative practice (IPCP) has repeatedly been linked to deficits in care and impaired patient-safety [1], [2], [3], [4]. Consequently, health policy makers across the globe have called for the use of IPCP to improve quality and safety of care and address the upcoming challenges to health services [5], [6], [7], [8]. Interprofessional education (IPE), occurring “when two or more professions learn with, from and about each other to improve collaboration and the quality of care” [9] is intricately linked with IPCP, but needs to be distinguished from interprofessional learning (IPL). IPL describes „…learning arising from interaction between members (or students) of two or more professions. This may be a product of IPE or happen spontaneously in the workplace or in education settings and therefore be serendipitous in nature“ [http://www.tandfonline.com/action/authorSubmission?journalCode=ijic20&page=instructions#.Vsrd3CwwfGE].

Interprofessional training wards (IPTW) describe a special form of undergraduate IPE/IPL/IPCP intervention which aim to enable students/trainees from different health professions to work together in interprofessional (IP) teams in order to manage the full responsibility for the medical treatment and rehabilitation of real life patients in an inpatient-hospital setting and at the same time gain interprofessional competencies [10]. Since their first description more than two decades ago in Sweden [11], multiple IPTWs have been established mainly in Scandinavia (reviewed in [10]) and other countries [12], [13], [14], [15], [16], [17], [18], [19], [20], [21], [22]. To date training wards have been piloted in Germany for medical students (Witten-Herdecke) [23], [24] and for nursing students, however an IPTW focussing on interprofessional competencies has not been established in Germany so far. Furthermore, IPTWs have been implemented across various medical disciplines ranging from internal medicine, geriatrics, obstetric to palliative care with a clear emphasis on orthopaedics [10]. To our knowledge none of the published IPTWs ever addressed the postoperative care of patients undergoing major abdominal surgery. 

Despite decades of IPE research astonishingly little is known about the impact of IPTWs on patient-relevant outcomes. First, this might be due to the very nature of IPTWs in which IPE, IPL and IPCP elements are closely intertwined as well as the lack of scientific rigor of studies within the interprofessional field [25], [26]. Existing studies within IPE have focused on learners satisfaction, acquisition of IP knowledge or modification of attitudes (level 1,2a/b outcomes according to the Joint Evaluation Team typology for outcomes of IPE [27]), rather than investigating behavioural changes (level 3), changes in organizational practice (level 4a) or benefits for patients (level 4b) [10]. Second, several authors have claimed that the atheoretical approach to IPE/IPL/IPCP research is responsible for these shortcomings and have called for more rigorous IP research by including psychological, behavioural and sociological models in the design and evaluation of IPE/IPL/IPCP [28], [29]. The multitude of learning theories proposed in IPE [30], [31], while showing on the one hand that IPE/IPL is truly located at the crossroad of multiple disciplines and fields, might at least partially explain this lack of rigorousness in the past. 

Aim of this paper is to describe the development of the IPTW curriculum and implementation of Germany´s first IPTW at the Department of Surgery at Heidelberg University Hospital (*Heidelberger Interprofessionelle Ausbildungsstation*, HIPSTA) based on best-practice examples from Sweden and with a theoretical underpinning. Furthermore, by using a transparent and generalizable methodology this project description may help others aiming to implement IPTWs. 

## Project description

We used the Kern cycle to develop our IPTW curriculum [32].

### Problem identification and general needs assessment

Health care systems around the world face immense challenges including the growing number of multi-morbid patients, demographic challenges, limitations of the health workforce, economic strains and advances in medical treatment [33]. IPCP occurring “when multiple health workers from different professional backgrounds provide comprehensive services by working with patients, their families, carers and communities to deliver the highest quality of care across settings” [5] has been identified as a key element to address these challenges. Shortcomings in collaboration between various health professions have repeatedly been linked to care failures, problems in work processes and patient safety [1], [2], [4]. Consequently health policy leaders across the globe have called for the use of IPCP as a key approach to improve the quality and safety of patient care [5], [6], [7], [8]. IPE and IPL are believed to be key elements to improve IPCP and patient care [5]. Therefore claims to incorporate and support IPE/IPL at under-, postgraduate and practice levels have been voiced by major health policy leaders [5], [6], [7], [8]. However, education and training of health care professions currently occurs predominately mono-professionally [34]. This discrepancy between the ideal approach of IPE, IPL and IPCP and the current approach of monoprofessionalism in training and daily practice has led to calls for improvement in the current “Masterplan Medizinstudium 2020” [35]. Finally chapter 8 of the national medical competency catalogue (*Nationaler Kompetenzorientierter Lernzielkatalog, NKLM* http.//www.nklm.de) states competencies medical students should acquire as members of a health care team (NKLM 8.1-8.4).

#### Targeted needs assessment

The special edition “Interprofessional Training” of the GMS Journal for Medical Education published in 2016 gives an overview of the current IPE landscape in Germany, Switzerland and Austria [36]). It reveals that IPE at an undergraduate level occurs only sporadically at different sites, is frequently optional rather than compulsory and lacks scientific rigor in its development and implementation. 

We identified three groups as targeted learners for an undergraduate curriculum in IPE/IPL/IPCP at our site in Heidelberg: medical students of the University of Heidelberg, nursing students of the Heidelberg Nursing School (*Akademie für Gesundheitsberufe Heidelberg*) and students of the bachelor programme “Interprofessional Healthcare” (*Interprofessionelle Gesundheitsversorgung*), IPHC. In order to identify the IPE contents with direct relevance to interprofessional collaborative patient care of the respective curricula we performed a curricular mapping of:

1. the clinical curriculum (Semester 5-10) at Heidelberg Medical School and
2. the curriculum of the qualification program “Gesundheits- und Krankenpflege” and
3. the curriculum of the B.Sc. Interprofessional Healthcare (IPHC).

Briefly, we were able to identify one single compulsory IPL unit for both student groups in the curricula of medical students and nursing students enrolled in the bachelor programme IPHC. Other IPL units were electives for medical students and compulsory for students enrolled in IPHC. Furthermore, while most of the teaching in the IPHC program has a clear focus on IP care, many are not delivered by interprofessional teaching teams. All professional curricula involve extensive compulsory workplace placements (*Praktika, Famulaturen, Praktisches Jahr* etc.). However all lack structured IP learning elements. How much IPE, IPL or IPCP occurs in these placements remains elusive and is highly dependent on the local workplace situation or supervisor/facilitator. Given the above outlined lack of IPCP in German hospitals the assumption that very little IPCP is taught or experienced by students seems not far-fetched. 

There is a noteworthy discrepancy at this point between targeted learners and learning environment on the one side and the goals and educational strategies on the other (see below) that calls for constructive alignment. As outlined above, the outcomes of IPE can be classified according to the Joint Evaluation Team Typology into 4 levels. It is feasible to design educational curricula for level 1/2 outcomes only and these basic IPE curricula (addressing level 1 or 2 outcomes) might be implemented early on during professional training. However, it is unclear when IPE should be implemented in the curriculum, with some authors arguing for an early implementation while others advocate a late implementation when students/trainees have gained some knowledge about their professional roles [37]. On the other hand, given their respective definitions, IPL and even more so IPCP imply an active interaction of health care professionals in a workplace setting. Therefore, by including IPL and IPCP aspects as goals/objectives into a curriculum, one also limits the learners to later stages of their training, when students/trainees have sufficiently advanced to interact in a workplace setting. As outlined below we aimed to do exactly that, i.e. build a curriculum that would incorporate IPCP aspects. Hence, we focused our curriculum to final year medical students (*Praktisches Jahr, PJ*) as well as final year nursing trainees, some enrolled in parallel to the bachelor programme IPHC.

#### Goals and objectives

We aimed to define broader goals and specific objectives for our curriculum. Substantial literature exists on this topic. First, core competencies for IPCP have been described in various frameworks [38], [39], [40]. Although differences between these frameworks exist, all emphasize

1. values/ethics for interprofessional practice,
2. roles/responsibilities,
3. interprofessional communication and
4. teams and teamwork as key components of successful IPCP.

The framework of the Interprofessional Education Collaborative (IPEC) defines a number of specific competencies for each of these domains [39]. Second, chapter 8 of the NKLM defines competencies for the medical doctor as team member. Given that the “*Masterplan Medizinstudium 2020*” puts a clear focus on competency-based curricula, we aimed to include these learning objectives. Third, given the intricate link between IPE, IPL and IPCP as defined above, we aimed to find objectives that incorporate aspects from all three areas. Furthermore, we believe that IPCP by definition implies cognitive processes from all six dimensions of the revised Bloom´s Taxonomy (remembering, understanding, applying, analysing, evaluating and creating) [41] which therefore have to be considered in the learning objectives. In addition, as IPCP implies a workplace setting, we aimed to incorporate workplace based objectives that would allow for assessment on the job. Consequently, objectives should be concise and practice-relevant, limited in number, specific and clear. We believe that Entrustable Professional Activities (EPA) are a perfect match for all of these requirements [42]. Finally, as IPE is more common in other countries we aimed to build on the experiences of others. To this end we visited the Karolinska University Hospitals between 7^th^ – 9^th^ December 2016. 

We defined two interprofessional objectives for our curriculum (see Attachment 1, Point A). In addition, when setting out to define educational strategies, this fed directly back to our goals and objectives and led to the definition of three interprofessional EPAs for our curriculum. We report the three interprofessional EPAs here for clarification (see Atttachment 1 , Point B). When building the EPA for interprofessional ward rounds we used the scaffold provided by Wolfe et al. and Berberat et al. [43], [44]. Finally, it is important to note that the interprofessional learning objectives were adopted from the Karolinska Institute, Stockholm, Sweden and the credit for this work goes entirely to them. 

#### Educational strategies

As pointed out above there is an intricate link between the goals and objectives and the educational strategies that led to the definition of the three EPAs. Within our group we strongly believe that an interprofessional real-life workplace setting is the best, if not only educational strategy that would allow for the integration of IPE, IPL and IPCP. 

However, IPTW is not a clearly defined educational strategy by itself and we thus aim to give a concise and clear description of our IPTW set-up and the reasons for doing so in order to clarify our approach. We searched the literature to identify previous IPTWs in order to generate ideas for our IPTW. We found multiple descriptions of IPTWs in different countries and specialities [10], [12], [13], [14], [15], [16], [17], [18], [19], [20], [21], [22]. However, the organisation and set-up of these IPTWs differs somewhat and was not always well described. Furthermore, all lacked a theoretical underpinning. In addition, we visited a number of IPTWs at Karolinska Institute, Stockholm, Sweden as best-practice examples to get insight into functioning IPTWs with a long successful track record. Finally, we aimed to incorporate psychological, educational and sociological IP theories to justify our HIPSTA approach [30]. 

**Figure 1:**
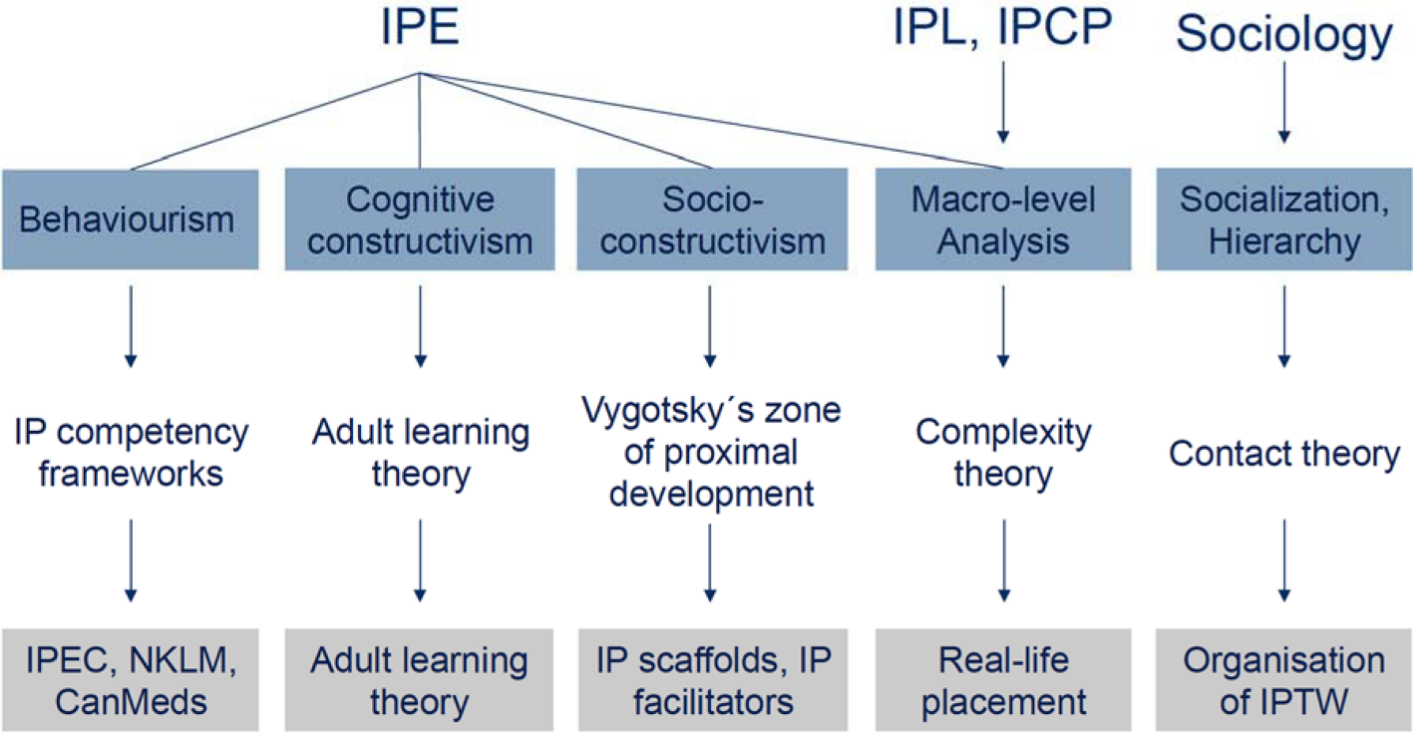
Schematic representation of the theoretical background of HIPSTA. IPE: interprofessional education. IPL: interprofessional learning. IPCP: interprofessional collaborative practice. IPEC: Interprofessional Education Collaborative. IP: interprofessional. NKLM: Nationaler Kompetenzorientierter Lernzielkatalog. IPTW: interprofessional training ward.

We condensed these three influences into the following educational strategy (overview Figure 1 (Fig. 1)). Addressing theory we decided on the one hand to opt for a behaviouristic approach by focusing on learning outcomes (see Attachment 1) rather than cognitive processes. In our opinion IP competency frameworks such as the IPEC framework we used for HIPSTA are inherently behaviouristic as they focus on measurable outcomes (see Figure 1 (Fig. 1)) [31]. We integrated this approach by using the IPEC competency framework [39], CanMeds and the NKLM in defining our EPAs and the organizational structure of our IPTW. 

On the other hand, we aimed to build HIPSTA around principles of adult learning theory which are constructivist in their origin [45], [46]. The assumptions of adult learning theory are that learners

1. “are independent and self-directing;
2. have accumulated experiences, which are rich resources for learning;
3. value learning that integrates with the demands of their daily lives;
4. are more interested in immediate problem-centered approaches than in subject-centred ones;
5. are more motivated to learn by internal as opposed to external drivers” [31].

By emerging advanced students/trainees in the care of real-life patients with complex surgical procedures, significant comorbidities and postoperative complications we aimed to address all of these points. 

The very definition of IPE, i.e. learning “with, from and about each other”, implies a social constructivist view whereby individual learning is mediated by the environment. Social constructivism, in contrast to cognitive constructivism, emphasizes how social encounters influence learners’ meanings and understanding [31]. Vygotsky used this social constructivist view to describe a zone of proximal development which is the difference between what a student can learn alone and what they can learn with the assistance of an “external other” [47]. This zone of proximal development varies in specific context but may be developed via teaching [48]. We addressed this issue by constructing an “external other” in two ways:

1. doctor and nurse facilitators who facilitate the interprofessional trainee team with their work on our IPTW. As shown on the Swedish best-practice examples these facilitators accompany the team, not necessarily being present all the time but within calling distance (EPA level 3);
2. providing written IP scaffolds for a set number of IP tasks (namely rounds, admission, discharge) that students may use during their self-responsible work.

O fourth, our own daily experience in working with surgical patients in a complex university setting, made us humble as to which degree we are really able to plan specific learning outcomes. To some degree we need to accept that on an IPTW IPL and IPCP is emergent and cannot be planned in all details. This seems to be best described by complexity theory as laid out by Cooper et al. [49]. 

Several authors have voiced their frustration with IPE in its tacit acceptance of hierarchy with health-care teams [28]. Numerous examples exist that some forms of IPE may indeed not foster IPCP, but rather reinforce professional stereotypes [50]. To address this issue we took into consideration data from contact theory in the organizational approach to HIPSTA [28], [51]. Contact theory states that individuals who are forced into intergroup interactions often experience negative results and positive intergroup contact requires equal status among participants [52]. As a result placement on HIPSTA is non-compulsory and students/trainees need to be at similar qualification levels. Furthermore, before placement, an IP is organized for all participants. 

Before starting on HIPSTA the new IP teams undergo an introductory day which covers the following topics:

1. principals of IPE, IPL and IPCP;
2. students and trainees establish feedback rules that are then printed and put up on the HIPSTA ward;
3. ward round training;
4. cardio-pulmonary resuscitation training (advanced life support) to prepare the IP team for medical emergencies.

Daily routine consists of ward rounds in the morning after which the IP teams meet and discuss the treatment and care plan for each patient (see Attachment 2). Both medical and nursing facilitators are present at this discussion, but remain in an observatory role. They can provide input as deemed necessary – not in the sense of giving solutions, but pointing towards possible solution strategies (e.g. where can the IP team find an answer to a certain problem? Which database could be consulted? Who would be the right person or department to contact etc.). Facilitators may give feedback to students during this time. Furthermore, learning areas of interest are identified and a student/trainee is asked to prepare a small (usually 5 min) teaching session/presentation for his fellow students/trainees during the afternoon hand-over. Consequently, presentations/tutorials are given by the students/trainees after handing over the patients in the afternoon meeting (see Attachment 2). The newly learned skills can then be applied directly to a certain case. If new case-based problems arise, another trainee/student is asked to prepare a tutorial for the following day. Facilitators may give their input if deemed necessary. As this learning is strictly case-based, topics may vary. However, judging from our experience, certain IP topics recur for each student cohort including breaking bad news (e.g. pathology results), postoperative mobilization, hygiene, wound care, anticoagulation, postoperative feeding, organizing post-hospitalization care/discharge management and others.

Additionally, guided self-reflections and structured feedback rounds are implemented once a week (usually on Fridays) to allow a reflection of the past week and identify goals for the upcoming week.

#### Organizational strategies

**Figure 2:**
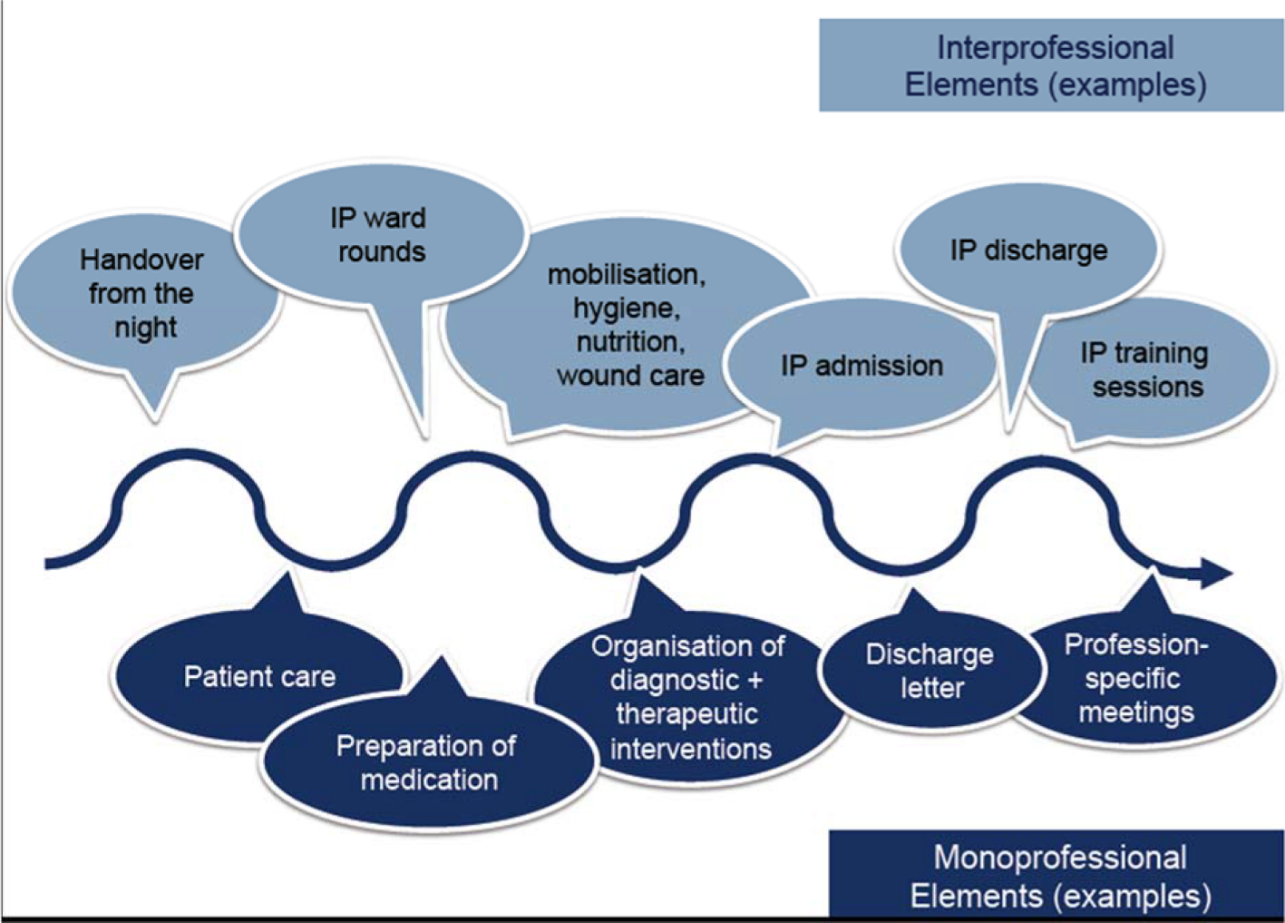
Schematic representation of the alternating mono- and interprofessional tasks on the HIPSTA ward.

In a next step we analysed medical and nursing routines in our hospital and aligned the two routines to define an interprofessional timetable. Furthermore, at this point, we assigned tasks to medical and nursing facilitators as well as to the two HIPSTA professions (medical students and nursing trainees). To increase patient centeredness and quality of care, two shifts (early and late) were defined to insure continuity of care for the patients for more than 15 hours per day. Based on our judgment of the complexity of cases and from the experiences in Stockholm, we decided that each HIPSTA team (one medical student and one nursing trainee) should be responsible for the care of 3 patients. Two HIPSTA teams work in parallel, resulting in the care of 6 patients. While working out this daily schedule it also became apparent that interprofessional and monoprofessional tasks alternate during the course of the day (see Figure 2 (Fig. 2)). Given the complex nature of our patients, the huge amount of professional skills our students need to learn alongside IP competencies and logistical constraints of the educational programmes we decided on 4 week-long placements. 

One of the most important daily appointments is the IP team meeting during which all members of our IPTW (early and late shift) and both facilitators (medical and nursing) are present (see Attachment 2). Interprofessional hand-over, communication, team roles and responsibilities can be assessed directly by the facilitators. It also serves to give feedback and educational input. 

In order to give students/trainees full immersion into the clinical work, special computer log-ins were generated that allow students/trainees the full range of professional activities including ordering examinations, prescribing medication, ordering lab values etc. with the restriction that these orders need to be confirmed electronically via their computer log-in or by hand signature by one of the medical facilitators. Only a handful of tasks have been identified that remain the sole responsibility of trained doctors/nurses e.g. obtaining informed consent from patients, blood transfusions etc. We used the guidelines given by the *“Möglichkeiten und Grenzen der Delegation ärztlicher Leistungen*” from the *Bundesärztekammer und Kassenärztliche Bundesvereinigung* (29.08.2008) as well as consultation with our legal department to define these tasks. 

Supervision is provided by the two facilitators (nursing and medical). While the nursing facilitator is present during the entire time, the medical/surgical facilitator is present only during morning rounds and for afternoon hand-overs. Doctors on the neighbouring ward can be consulted at all times. Likewise, the medical facilitator can be consulted by phone at all times.

#### Implementation 

In order to implement out IPTW project we formed a steering group consisting of members from all relevant professions and involved institutions:

1. surgeons from the Department of General, Visceral and Transplantation Surgery (ALM, TK, PP);
2. head of nursing and clinical nurse specialists from the Department of Surgery (GM, BTH, JS);
3. members of the Heidelberg Nursing School (BG);
4. members of the bachelor program IPHC (CM, AM, JM);
5. student representatives from the Heidelberg University Medical School (CF, AB, JC) and
6. trainee representatives from the Heidelberg Nursing School (LM, AC).

After coming together the project was planned, designed and implemented by this steering group. In a first step a funding application was written for the Robert-Bosch-Stiftung funding programme *“Operation Team – Interprofessionelles Lernen in den Gesundheitsberufen*“. Acquiring funding made subsequent steps easier as money was available for travelling and for financing the nursing facilitators. Furthermore, it committed the group to a strict time schedule. 

In order to plan the implementation of the curriculum, a SWOT (Strength, Weaknesses, Opportunities and Threats) analysis was performed (not shown) and a Gantt-chart was drawn to plot tasks on a time axis (see Attachment 3). Most importantly, we performed a RASCI analysis (Responsibility, Approval, Support, Consultation, Information) (see Attachment 4). Gantt-chart and RASCI table could be useful to readers interested in establishing an IPTW at their site. 

We would like to point out three specific problems we encountered while implementing our IPTW that we believe are noteworthy. First, it took unexpectedly long to establish the necessary IT log-in for the students. To really work self-responsibly IPTW students/trainees basically need the same rights as doctors/nurses, while at the same time legal aspects have to be considered. At our site this necessitated the creation of special IPTW student accounts. Furthermore, we created a number of new, interprofessional documents (e.g. for ward round documentation or admission) that required implementation in our hospital IT system. Hence, early integration of the IT service is of utmost importance. Second, to create a “safe place with a space for learning” [53], IPTW students/trainees need their own room (HIPSTA room) and computer access. This room is essential for IP team meetings and daily work, but was difficult to organize in a busy, space-limited hospital setting. Third, the coordination of the different professional curricula is challenging. Ensuring continuous placements of an interprofessional team over time requires compromises and goodwill from all involved professions. 

## Results

In short, we succeeded in establishing HIPSTA successfully in April 2017 and have run our IPTW since then with 11 cohorts (more than 90 students/trainees with 4 week-placements each). Students/trainees run the IPTW in two shifts (early and late), alternating each week. Students/trainees work together in interprofessional (IP) teams and manage the full responsibility for the medical treatment and rehabilitation of real life patients. Profession-specific facilitators support the students throughout the day. Attachment 2 gives an overview over the daily routine on HIPSTA.

While initially we aimed to exclude some patients with very complex postoperative care protocols from our IPTW (e.g. following liver transplantation) this limitation has since been lifted as trust and capabilities of the HIPSTA teams were self-evident and became apparent within our hospital. 

There are numerous positive examples of successful IPL and IPCP on HIPSTA. One of the most striking is the motivation of the IP teams. For example, students/trainees have started to improve and expand a set of interprofessional standard operation procedures (SOP) incorporating the perspective of their respective professions. This SOP collection has expanded over time and currently includes more than 40 protocols. 

IP teams showed admirable creativity in communicating therapeutic, diagnostic and rehabilitative procedures to patients and their families. For example, IPTW teams started to write short-discharge reports in lay language for patients and their families besides the usual medical discharge report to enable patients to better understand their hospitalisation, surgery, planned adjuvant treatment and rehabilitation.

Furthermore, as could be expected, several IP teams were confronted with medical and surgical emergencies during their placement including pulmonary thromboembolism, anastomotic leackage, stroke and myocardial infarction. In all cases students/trainees identified the problem quickly and responded correctly. Given the constant supervision by the nursing facilitator and the quick involvement of the medical facilitator/doctors from the neighbouring ward, patient safety was not compromised at any time. Although objective data is lacking, the tight care of students/trainees on HIPSTA might actually have speeded treatment and emergency interventions compared to a conventional ward. 

Satisfaction of students, facilitators and patients was high, but further evaluation is pending. The next step in the Kern cycle is evaluation and feedback. A full analysis of results is beyond the scope of this paper. We are currently evaluating the first HIPSTA cohorts and will report the results in the future. 

## Discussion

Here we describe the successful establishment of an IPTW at the Department of Surgery at Heidelberg University Hospital. To our knowledge this is the first IPTW in Germany and the first in abdominal surgery worldwide. HIPSTA is running successfully since April 2017. 

There are two main objectives we tried to achieve with this publication:

1. to give a clear description of how we managed to establish our IPTW that may serve as a blueprint for others aiming to establish an IPTW;
2. to describe how a practice- and theory-guided design can be successfully transferred into clinical practice.

The early involvement of all professions seems to be key to success. At the same time a dedicated steering group with a limited number of representatives from all involved professions is necessary to plan and implement an IPTW. We have highlighted some of the major challenges in the implementation section of this paper. Since initiation only minor modifications to our planned curriculum were necessary showing the success of our interprofessional approach. Although numerous examples show that IPTW can be established without a clear theoretical background, we aimed to incorporate theory in the design and set-up of our IPTW as outlined in Figure 1 (Fig. 1). This is an important aspect as IPE in general and IPTWs have been criticised for being atheoretical [28], [29]. 

As numerous IPTWs have been established in various fields of medicine and across multiple countries [10], [12], [13], [14], [15], [16], [17], [18], [19], [20], [21], [22], we conclude that there are no principal limitations to the application of IPTWs. However, if IPTWs really improve IPE, IPL, CP and ultimately patient care remains elusive. Although the set-up was not interprofessional, it has been shown recently that strengthening “supported active participation” of medical students in patient care resulted in superior patient- physician/student-interaction and quality of care as perceived by patients on training wards in Witten-Herdecke when compared to matched pairs of the same clinical specialty from the same hospital or from nationwide hospitals [54]. 

Furthermore, we would like to emphasize the importance of the facilitators working on an IPTW. As there is no formal training to become an IPTW facilitator, commitment and self-directed interprofessional competency development is needed by all people involved. Astonishingly little research has been done in this area [15]. As outlined above we regard the IP facilitators to have a central role that facilitate the IP learning process of the team, but how this is brought about remains elusive and warrants further investigation.

There are several limitations to our project. First, numerous IPTWs have been described before mainly in Scandinavia, the UK, Canada and Australia [10], [12], [13], [14], [15], [16], [17], [18], [19], [20], [21], [22]. Therefore our project is hardly new. Indeed, previous publications as well as the personal visit to some of the oldest active IPTWs at Karolinska University gave us valuable insights for our project. However, to our knowledge this is the first IPTW project description based on theoretical background. Second, this is a single-centre description only and the conclusions we have drawn might not be applicable in other clinical settings. Third, this is a mere project description without a qualitative or quantitative evaluation of IPE, IPL or IPCP. Therefore, it remains elusive if our IPTW really improves IPCP and subsequently patients care (level 4 outcome according to Joint Evaluation Team typology) but it could be studied in the future with methodologically sound clinical trials. 

## Conclusions

We succeeded in establishing and running an IPTW in Germany involving complex multi-morbid patients undergoing major abdominal surgery at the Department of Surgery at Heidelberg University Hospital. An interprofessional steering group can successfully implement an IPTW using a structured curricular approach to address educational aspects and project management tools to address organizational issues. Design and set-up of an IPTW should be guided by practice and theory. 

## Abbreviations

- AfG: *Akademie für Gesundheitsberufe gGmbH * (Heidelberg School of Nursing)
- EPA: Entrustable Professional Activity
- HIPSTA: *Heidelberger Interprofessionelle Ausbildungsstation* (Heidelberg Interprofessional training ward)
- IP: Interprofessional
- IPCP: Interprofessional collaborative practice
- IPEC: Interprofessional Education Collaborative
- IPHC: Bachelor programme “Interprofessional Healthcare”
- IPE: Interprofessional education
- IPL: Interprofessional learning
- IPTW: Interprofessional training ward
- NKLM: Nationaler Kompetenzorientierter Lernzielkatalog
- PJ: *Praktisches Jahr* (final year of medical school)
- RASCI: Responsibility, Approval, Support, Consultation, Information
- SOP: standard operation procedures

## Funding

Implementation of HIPSTA is funded by the Robert-Bosch-Stiftung, „Operation Team – Interprofessionelles Lernen in den Gesundheitsberufen“ (funding number: 32.5.A381.0026.0). No financial support was given other than this funding. There are no restrictions on publications and no conflicts of interest. The idea for HIPSTA was conceived, designed and implemented independent of any financial funder. The funder and the HIPSTA study group are independent. 

## Acknowledgement

We would like to cordially thank Rene Ballnus and the interprofessional training teams at Karolinska University Hospital Stockholm for their hospitality during our visit from 7^th^-9^th^ December 2016 and their willingness to discuss and share their profound knowledge and experience on interprofessional education and training wards with us. 

## Competing interests

The authors declare that they have no competing interests. 

## Supplementary Material

Attachment 1: A. Interprofessional learning objectives (Karolinska Institute, Stockholm; Rene Ballnus). B. Interprofessional Entrustable Professional Activities (EPAs).

Attachment 2: Summary of daily routine on HIPSTA.

Attachment 3: Gantt-chart for the establishment of the HIPSTA IPTW.

Attachment 4: Responsibility, Approval, Support, Consultation, Information (RASCI) scheme of HIPSTA. *see Gantt-chart (see Attachment 3). AfG: Akademie für Gesundheitsberufe Heidelberg. IPE: interprofessional education.
