# Supplementary material for: Heidelberger Interprofessionelle Ausbildungsstation (HIPSTA): a practice- and theory-guided approach to development and implementation of Germany’s first interprofessional training ward
Source: GMS J Med Educ. 2018 Aug 15;35(3):Doc33. doi: 10.3205/zma001179 (PMC6120150; doi:10.3205/zma001179)
Supplement: Attachment 1: A. Interprofessional learning objectives (Karolinska Institute, Stockholm; Rene Ballnus). B. Interprofessional Entrustable Professional Activities (EPAs). [file JME-35-33-s-001.pdf]

A

Learning objective	Pass	Fail
Together with the team, using an ethical approach, the student shall be able to analyse and meet the patient's needs, and evaluate the patient's treatment, nursing care and rehabilitation	Is active within the team, seeks cooperation and shows respect for colleagues. Takes active part in discussions and reflections.<br><br>Takes responsibility for the patients' treatment; makes appropriate suggestions regarding nursing, medical care or rehabilitation; takes responsibility for his/her part of the work.<br><br>Is able, in collaboration with the team, to prioritise and plan work load and allocation of time.	Often needs support and encouragement in order to cooperate with the team, and displays insufficient respect for co-workers on the team. Display shortcomings in participation in discussions and reflections.<br><br>Has difficulty taking responsibility for the patients; has few adequate suggestions for nursing, medical care, or rehabilitation.<br><br>Has difficulty taking responsibility for planning workload and time in cooperation with the team.
The student shall be able to reflect on his/her own skills and those of other professions for increased patient safety, and demonstrate the ability to communicate and collaborate with patients and their families and with other professions	Knows the patients' needs and rights well, and represents them. Respects the patient's integrity. Includes the patient and family as cooperation partners.<br><br>Shows an active interest in learning from, with and about the other professions; reflects on and integrates this knowledge in his/her own professional development<br><br>Gives relevant and clear information within the team and to patients, family and other personnel<br><br>Is able to summarise the treatment period orally/in writing, and takes the experience of the entire team into consideration.	Shows insufficient understanding of the patient's needs and insufficient respect for the patient's integrity. Displays considerable deficiencies in communicating with patients and families.<br><br>Needs help in making use of the opportunities to learn from, with and about the other professions; focuses on his/her own profession to a large extent; has insufficient abilities in reflecting on and integrating knowledge.<br><br>Information/instructions to team members, patients, families and other personnel is frequently unclear, insufficient, or too detailed.<br><br>Displays insufficient ability in summarising the treatment period orally/in writing, and insufficient consideration of the entire team's experience.

B

Type of EPA	Interprofessional surgical ward round
Discipline	PJ students, nursing trainees
Summary	Conduct interprofessional ward rounds of postoperative patients on HIPSTA and jointly prepare an interprofessional plan for further diagnostic, therapeutic, nursing and rehabilitative treatment of the patient.
Frequency	Daily

NKLM/CanMeds competencies	Medical expert 5.1, 5.2.1.3, 5.3, 5.4<br>Communicator 7.1, 7.2, 7.3, 7.4, 7.6<br>Collaborator 8.1.1, 8.1.2, 8.2.1, 8.2.2, 8.2.3, 8.3<br>Leader/manager 10.1, 10.2.1.1, 10.6, 10.7, 10.8, 10.10<br>Professional 11.1.1, 11.1.2, 11.1.4, 11.1.5, 11.2.3, 11.3.2, 11.4
Superior knowledge and skills	1. Planning of diagnostic and therapeutic procedures<br>2. Leadership competencies<br>3. Empathy<br>4. Ability to respond to complications, interruptions and difficult situations<br>5. Organizational skills and self-management<br>6. Professionalism<br>7. Teaching and learning skills
Necessary knowledge and skills	• Knowledge about the nursing, medical and surgical history and the current diagnoses of the patient<br>• Knowledge of the current nursing, medical, surgical treatment plan<br>• Knowledge of possible postoperative complications<br>• Knowledge of the medical and nursing management of postoperative patients in an organized and timely manner<br>• Knowledge of postoperative pain, wound and mobilization management<br>• Knowledge of discharge management and rehabilitation<br><br>• Ability to independently structure, plan and organize ward rounds as a team.<br>• Ability to ask focused questions during rounds.<br>• Ability to provide patients, relatives and team members with comprehensive information on treatment, diagnosis and procedures.<br>• Ability to collect information from patients, relatives and other team members from different sources (interview, files, clinical examination) and incorporate them into the treatment plan.<br>• Ability to establish a trustful doctor - nurse-patient relationship through appearance, gestures and behaviour.<br>• Ability to identify and resolve conflicts within the doctor - nurse-patient relationship.<br>• Ability to show empathy in the doctor - nurse-patient relationship.<br>• Ability to develop an interprofessional treatment plan that adequately integrates surgical, medical, nursing, ethical and patient-specific aspects.<br>• Ability to jointly implement rounds and the resulting interprofessional treatment plan as a team, taking into account the time and resources available.<br>• Ability to reflect on one's own strengths and weaknesses and the consequences of one's own actions, and to know when to consult a facilitator.<br>• Ability to remain calm in difficult situations and to act purposefully, focused and quickly as a team.<br>• Ability to react appropriately to interruptions.<br>• Ability to seek and acquire new knowledge based on ward rounds<br>• Ability to document rounds and the resulting treatment plan together as a team
Comeptence level (aim)	• Level 3 = practice EPA with indirect (re-active) supervision
Assessment	• Assessment of ward rounds by nursing and medical facilitators.<br>• Assessment of the daily interprofessional team meetings and handovers by nursing and medical facilitators.<br>• Assessment of the interprofessional ward round documentation by medical and nursing facilitators.<br>• Assessment of the incorporation of new HIPSTA team members by medical and nursing facilitators.<br>• Patient feedback questionnaires.

Type of EPA	Interprofessional patient admission
Discipline	PJ students, nursing trainees
Summary	Perform an interprofessional preoperative admission of a patient to a surgical ward
Frequency	Daily
NKLM competencies	Medical expert 5.1, 5.3<br>Communicator 7.1, 7.2, 7.3, 7.4<br>Member of a team 8.1.1, 8.1.2, 8.2.1, 8.2.2, 8.2.3, 8.3<br>Leader/manager 10.6, 10.7, 10.8, 10.10<br>Professional actor 11.1.1, 11.1.2, 11.1.4, 11.1.5, 11.2.3, 11.3.2, 11.4
Necessary knowledge and skills	• Knowledge of the underlying disease, the planned surgical treatment and the necessary preoperative work-up<br>• Knowledge about indications and contraindications for surgery<br>• Knowledge how missing information can be obtained.<br>• Knowledge how the individual patient situation, the planned surgery and his/her comorbidities may adversely effect the pre-, intra- and postoperative course and the rehabilitation of the patient.<br><br>• Ability to perform an interprofessional history in a, focused and complete manner, taking into account the individual patient situation and medical as well as nursing aspects.<br>• Ability to perform a focused physical examination<br>• Ability to document the medical history and physical examination.<br>• Ability to plan necessary preoperative procedures interprofessionally in the team and to initiate the necessary examinations.<br>• Ability to present the patient's medical history as well as the surgical indication concisely to one's own and to other professions.<br>• Ability to provide patients, relatives and team members with comprehensive information on the disease and surgical indication.<br>• Ability to reflect on one's own strengths and weaknesses and to know when to ask a facilitator for advice.
Comeptence level (aim)	• Level 4 = may perform EPA on their own
Assessment	• Assessment of admissions by medical and nursing facilitators.<br>• Assessment of interprofessional admission documents<br>• Assessment of the case presentation in the daily indication meetings<br>• Assessment of the case presentation in the interprofessional team meetings by medical and nursing facilitators.

Type of EPA	Interprofessional discharge management
Discipline	PJ students, nursing trainees
Summary	To manage the discharge of a surgical patient together as an interprofessional team from the acute care setting to the subsequent health care sector (e.g. home, nursing home, rehabilitation centre) considering the individual needs of the patient as well as the intersectoral characteristics of the health care system
Frequency	weekly
NKLM competencies	Medical expert 5.1, 5.2.1.3, 5.3, 5.4<br>Communicator 7.1, 7.2, 7.3, 7.4, 7.6<br>Member of a team 8.1.1, 8.1.2, 8.2.1, 8.2.2, 8.2.3, 8.3<br>Leader/manager 10.1, 10.2.1.1, 10.6, 10.7, 10.8, 10.10<br>Professional actor 11.1.1, 11.1.2, 11.1.4, 11.1.5, 11.2.3, 11.3.2, 11.4
Necessary knowledge and skills	• Knowledge about the current medical history, diagnosis and treatment of the patient<br>• Knowledge of the outpatient medical, nursing and rehabilitation treatment plan<br>• Knowledge about ambulatory care, discharge management, rehabilitation and the structure of the health care system

	• Ability to develop a post-discharge rehabilitation and treatment plan together with the team, taking medical, nursing, ethical and patient-specific aspects into account<br>• Ability to plan and implement a post-discharge treatment and rehabilitation plan while maintaining meaningful time and resource management<br>• Ability to communicate the post-discharge rehabilitation and treatment plan with patients, relatives, caregivers, primary care physicians, and other involved personnel.<br>• Ability to train patients and relatives in necessary postoperative procedures and to provide understandable information about the post-discharge treatment and rehabilitation plan<br>• Ability to reflect on one's own strengths and weaknesses and to know when to ask a facilitator for advice
Comeptence level (aim)	• Level 4 = may perform EPA on their own
Assessment	• Assessment of interprofessional discharge management and discharge conversations by medical and nursing facilitators.<br>• Assessment of the daily interprofessional team meetings and handovers by medical and nursing facilitators.<br>• Assessment of interprofessional discharge documentation by medical and nursing facilitators.<br>• Feedback of patients and families during rounds and discharge talks.<br>• Patient feedback questionnaires
