# Supplementary material for: Heidelberger Interprofessionelle Ausbildungsstation (HIPSTA): a practice- and theory-guided approach to development and implementation of Germany’s first interprofessional training ward
Source: GMS J Med Educ. 2018 Aug 15;35(3):Doc33. doi: 10.3205/zma001179 (PMC6120150; doi:10.3205/zma001179)
Supplement: Attachment 2: Summary of daily routine on HIPSTA. [file JME-35-33-s-002.pdf]

HIPSTA Daily Routine

Time	Nursing Facilitator	Nursing Trainee	Medical Student	Medical Facilitator	
6.50 -7.00	Handover from night shift			Consultation as necessary	Early shift
7.00 -7.20	• Accompany patients to operation room and/or dialysis		• Obtain blood samples		
		• Obtain Blood samples			
7.20 -8.00		• Patient care according to care plan	• Patient examination		
		Patient examination			
7.25 -8:00		Early meeting			
8.00-8.15		• Breakfast<br>• Assess nutritional status<br>• Support patients	• Prepare for ward rounds<br>• Revise to-do-list<br>• Medical consultation		
8.15 -9.00	Interprofessional Ward Round with consecutive IP discussion and treatment plan;<br>Distribution of topics for afternoon presentations/tutorials (case-based)				
9.00 -9.30		• IP Care plan<br>• Written IP documentation of ward rounds		Instructions as needed	
	• Discussion of the objectives of daily care plan		• Work-off ToDo list from ward rounds<br>• Obtain necessary information for treatment plan (EbM)<br>• Plan examinations and consultations		
9.30 - 10.00	Break				
	• Patient care according to care plan		• Write medical reports		
10.00 - 10.15		• Write nursing reports			
		• Interprofessional discussion<br>• IP discharge planning for all patients<br>• IP Discharge interviews			

10.15 -<br>11.00		• Admission of new patients<br>• Writing of IP admission notes			
	• Supervision and instructions as needed	• Nursing history, assessment and care planning	• Treatment plan<br>• Examinations and consultations		
11.00 -<br>12.30	• Supervision and instructions as needed	• Patient Care	• Treatment plan<br>• Examinations and consultations		
12.00		• Lunch			
		• Assess nutritional status			
12.30 -<br>13.00	• Documentation				
	Prepare for hand over				
13.00 -<br>14.30	Interprofessional handover and case discussion, case-based learning, IP tutorials/presentations, guided self-reflection				
14.30 -<br>15.00		• Patient care, assessment	• Write medical reports<br>• Obtain necessary information (EbM) about diseases, treatment<br>• Preparation of informed consent	• Accompany "Kurvenvisiste"<br>• Consultation as needed<br>• Obtain Informed consent	Late shift
15:00 –<br>15:45					
15.00		• Prepare patients for surgery	• Radiology conference and indication meeting		
from<br>15.00		• Patient care	• Examination of post-OP patients		
from<br>16.00		"Kurvenvisiste"			
form<br>16.30		• Set up drugs			
from<br>17.00		Prepare discharge			

18.00		• Dinner			
18.00 - 18.30		Break			
from 18.30		• Prepare patients for surgery	• Prepare preoperative patients for surgery		
from 19.00		• Patient care			
20.00		• Documentation			
21.00		Hand over to night shift			

Supplement 2. Summary of daily routine on HIPSTA.
