# Supplementary material for: Heidelberger Interprofessionelle Ausbildungsstation (HIPSTA): a practice- and theory-guided approach to development and implementation of Germany’s first interprofessional training ward
Source: GMS J Med Educ. 2018 Aug 15;35(3):Doc33. doi: 10.3205/zma001179 (PMC6120150; doi:10.3205/zma001179)
Supplement: Attachment 3: Gantt-chart for the establishment of the HIPSTA IPTW. [file JME-35-33-s-003.pdf]

	Schedule																		
	Year 1												*Year 2*						
Milestones	1	2	3	4	5	6	7	8	9	10	11	12	1	2	3	4	5	6	7
**1. Defining the organisational structure of the IPTW**																			
a.) Analysis of daily routines	■																		
b.) Agreeing on a common IP daily IPTW routine		■																	
c.) Distribute of task ("who does what, when on HIPSTA?")			■	■															
d.) Visiting a best-practice example					■														
e.) clarification of legal aspects					■	■	■												
**2. Designing and integrating educational measures**																			
a.) Design of IP scaffolds and SOPs						■	■												
b.) Design of IP training curriculum							■	■											
c.) Integration of HIPSTA into curricula						■	■	■											
**3. Administrative , curriculare and organisational implementation**																			
a.) training of facilitators								■											
b.) organisation of rooms and materials			■	■	■	■													
c.) set-up of IT measures				■	■	■	■	■											
**4. Realisation**																			
a.) trial run									■	■	■	■	■	■	■	■	->	->	
**5. Evaluation**																			
a.) Plan IPTW evaluation						■	■	■											
b.) Evaluation									■	■	■	■	■	■	■	■	->	->	
