# Supplementary material for: Heidelberger Interprofessionelle Ausbildungsstation (HIPSTA): a practice- and theory-guided approach to development and implementation of Germany’s first interprofessional training ward
Source: GMS J Med Educ. 2018 Aug 15;35(3):Doc33. doi: 10.3205/zma001179 (PMC6120150; doi:10.3205/zma001179)
Supplement: Attachment 4: Responsibility, Approval, Support, Consultation, Information (RASCI) scheme of HIPSTA. *see Gantt-chart (see Attachment 3). AfG: Akademie für Gesundheitsberufe Heidelberg. IPE: interprofessional education. [file JME-35-33-s-004.pdf]

Responsibility	Argument	When*	How
Medical education coordinator	• Interprofessionality<br>• *Masterplan Medizinstudium 2020*<br>• IPE increasingly important (WHO guidelines)<br>• Cooperation<br>• Student/trainee satisfaction<br>• Clinical usefullness<br>• Money (when funded)<br>• Flagship project	Milestone 1	- Personal meeting<br>- Presentation
PJ coordinator	• Improved training, education<br>• Preparation for future work as a doctor<br>• Self-Responsibility<br>• *Masterplan Medizinstudium 2020*	Milestone 1	- Personal meeting<br>- Presentation
Nursing management	• Interprofessionality<br>• Cooperation<br>• Clinical Meaning<br>• Money (when funded)<br>• Flagship project	Milestone 1	- Personal meeting<br>- Presentation
Nursing Practice instructor	• Strengthening of IPE and cooperative practice<br>• Increased interaction with nurses and doctors<br>• Opportunity to strengthen nursing aspects/roles<br>• Opportunity to implement own ideas	Milestone 1	- Personal meeting<br>- Presentation
School of nursing	• Strengthening of IPE and cooperative practice Improved training<br>• Possibility to implement own ideas<br>• Enhanced interaction with hospitals, students, doctors	Milestone 1	- Personal meeting<br>- Presentation
Bachelor programme IP health care	• Practical experience in the transposition and implementation of IPE<br>• Scientific support and research opportunities<br>• Extending of IP course units in trainings<br>• Strengthening of IPE in Medical Faculty and AfG	Milestone 1	- Personal meeting<br>- Presentation<br>-Participation
Student council of medicine	• Improved training<br>• Preparation for future work as a doctor<br>• Self-responsibility<br>• IPE and coorative practice	Milestone 1	- Personal meeting<br>- Presentation<br>- Participitation

Commissioner of trainees of AfG	• Improved training<br>• Preparation for future work as a nurse<br>• Responsibility<br>• IPE and coorative practice	Milestone 1	- Personal meeting<br>- Presentation<br>- Participitation
**Approval**	**Argument**	**When***	**How**
Dean and academic dean	• Flagship project<br>• IPE increasingly important (WHO guidelines)<br>• *Masterplan Medizinstudium 2020*	Milestone 1	- Personal meeting<br>- Presentation
Managing clinic director	• Flagship project<br>• Attract future trainees in surgery and nursing<br>• Improved patient care<br>• Enhanced student trainee/Interest in surgical department<br>• IPE increasingly important (WHO guidelines)<br>• *Masterplan Medizinstudium 2020*	Milestone 1	- Personal meeting<br>- Presentation
Management level of department	• Improved patient care<br>• Enhanced student/trainee Interest in department<br>• IPE increasingly important (WHO guidelines)<br>• *Masterplan Medizinstudium 2020*	Milestone 3	- Presentation in morning meeting/grand rounds<br>- Kick-off Meeting
Administration	• Improved patient care<br>• Enhanced student/trainee Interest in department<br>• IPE increasingly important (WHO guidelines)<br>• Potential cost-effectiveness (reduced length of stay, publications)	Milestone 3	- Personal meeting<br>- Presentation
Staff council	• Integration of trainees in project<br>• Protection of operational and legal requirements<br>• Continuous supervision available	Milestone 3	- Personal meeting<br>- Presentation
University committees (e.g. faculty council)	• Flagship project<br>• Secure trainees in surgery and nursing<br>• Improved patient care<br>• IPE increasingly important (WHO guidelines)<br>• Protection of operational and legal requirements<br>• Continuous supervision available	Milestone 3	- Personal meeting<br>- Presentation
**Support**	**Argument**	**When***	**How**
Surgical and AfG IT department	• Approval and support by directors<br>• Being part of pilot project	Milestone 3	- Email<br>- Presentation

	• Opportunity to implement and test new IT solutions in a pilot setting		- Phone conversation
University IT centre	• Opportunity to implement and test new IT solutions in a pilot setting	Milestone 3	- Email<br>- Presentation<br>- Phone conversation
Legal department of University Hospital HD	• Approval und Support by directorate and clinic management<br>• Part of flagship project<br>• Publications	Milestone 1	- Personal conversation<br>- Presentation<br>- Written transcripts of HIPSTA activities
Sponsors	• Stringent funding proposal<br>• Unique project in Germany<br>• IP character	Milestone 1	Funding proposal
**Consultation**	**What**	**When***	**How**
IPTW experts	• Visit a functioning IPTW<br>• “Reality check” of own concept	After milestone 1	- Visit an IPTW<br>- Email contact<br>- personal contact at meeting
**Information**	**What**	**When***	**How**
All medical students of University of HD	• Summary of project	After milestone 4	Email newsletter,<br>Information event,<br>PJ-day
All trainees and students of the nursing school	• Summary of project	After milestone 3	- Email newsletter,<br>information event, inauguration event
All employees of University Hospital HD	• Summary of curriculum	After milestone 4	Report on “Klinikticker”/ Homepage
Expert audience	• Results of evaluation	After milestone 5	- Presentation<br>- Publication
